# 6-Methoxyflavone induces S-phase arrest through the CCNA2/CDK2/p21CIP1 signaling pathway in HeLa cells

**DOI:** 10.1080/21655979.2022.2047496

**Published:** 2022-03-04

**Authors:** Chaihong Zhang, Yuchong Quan, Lijuan Yang, Yingying Bai, Yongxiu Yang

**Affiliations:** a The First Clinical Medical College of Lanzhou University, Lanzhou, Gansu, China; b Key Laboratory of Gynecological Oncology of Gansu Province, Lanzhou, Gansu, China; c College of Basic Medicine, Dalian Medical University, Dalian, Liaoning, China; d Department of Obstetrics and Gynecology, First Hospital of Lanzhou University, Lanzhou, Gansu, China

**Keywords:** 6-methoxyflavone, cell cycle, cervical cancer, network pharmacology, molecular docking

## Abstract

This study aimed to elucidate the specific anticancer mechanism of 6-methoxyflavone in HeLa cells. A total of 178 putative targets of 6-methoxyflavone were obtained from the PharmMapper database. Microarray analyses, transcriptome sequencing analyses, functional enrichment analyses, and gene set enrichment analyses were performed to preliminarily explore the roles and mechanisms of the 178 targets in cervical cancer. Cell counting kit-8, cell cycle assays, polymerase chain reactions, and western blotting were used to clarify the mechanism of action of 6-methoxyflavone. Molecular docking and noncovalent interaction analyses were performed to further confirm the mechanism of action in three-dimensional structures. Functional enrichment analyses and gene set enrichment analyses indicated that high mRNA expression of cyclin A2 (CCNA2) and cyclin-dependent kinase 2 (CDK2) stimulated cell cycle progression in cervical cancer. Cell proliferation and cycle assays, transcriptome sequencing, polymerase chain reactions, and western blotting revealed that 6-methoxyflavone inhibited HeLa cell proliferation and induced S-phase arrest via the CCNA2/CDK2/ cyclin-dependent kinase inhibitor 1A (p21CIP1) pathway. Molecular docking and noncovalent interaction analyses showed that 6-methoxyflavone had the strongest affinity toward, inhibitory effect on, and noncovalent interactions with CDK2, and that the combination of CDK2 and CCNA2 enhanced these effects. An analysis of clinical characteristics showed that 6-methoxyflavone might be related to six clinicopathological parameters of cervical cancer patients. 6-Methoxyflavone induces S-phase arrest in HeLa cells via the CCNA2/CDK2/p21CIP1 pathway.

## Graphical Abstract

**Figure:**
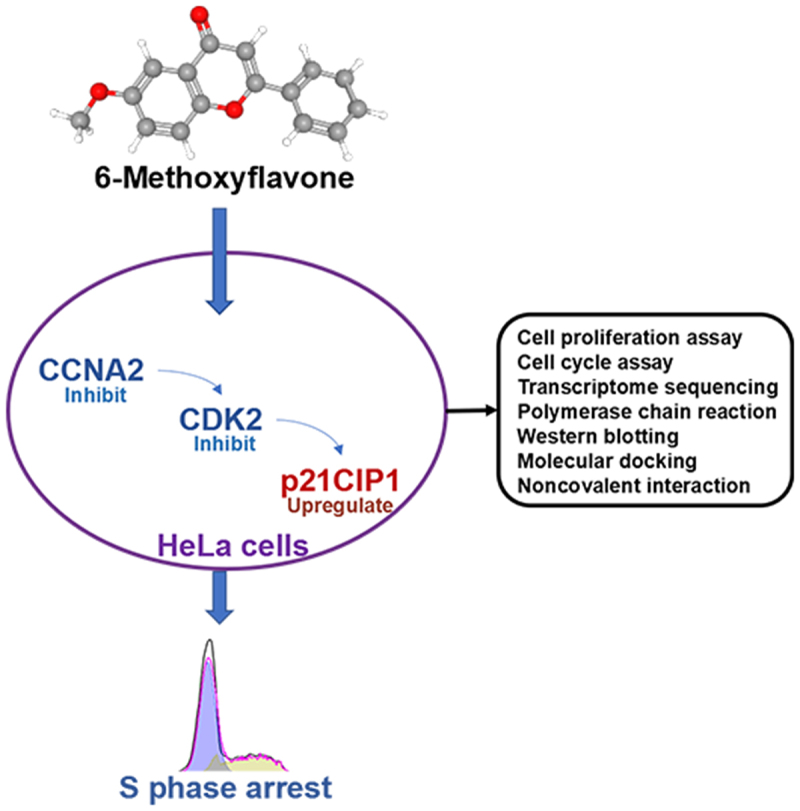

## Introduction

Cervical cancer, including cervical adenocarcinoma, is a prominent threat to the health of women. The incidence [1], therapeutic tolerance rate [2], recurrence rate [3], probability of pelvic lymph node involvement [4], possibility of ovarian invasion [5] and distant metastasis rate [6] of cervical adenocarcinoma remain high. Chemotherapy is an important complement to surgical treatment for cervical cancer. However, there are still challenges regarding drug sensitivity, targeting, toxicity, and resistance. Hence, low-toxicity and high-sensitivity drugs and in-depth studies of their mechanisms of action are urgently needed.

Various Chinese herbal decoctions have been used to treat tumors for thousands of years. Plant-derived anticancer drugs such as paclitaxel, camptothecin series, podophyllotoxin, harringtonine, and turmeric extract have been approved for commercial sale for decades. Traditional Chinese medicine and its active ingredients have an increasingly important role in cancer treatment. 6-Methoxyflavone is derived from the Chinese herbal medicine Imperata Cylindrica and is an effective ingredient of Imperatae rhizoma. Previous studies of 6-methoxyflavone have shown a wide range of allosteric modulatory effects [7], immunological regulation effects [8], antiproliferation and anticancer effects [9], antinociceptive effects [10], and anti-inflammatory effects [11]. A chemotherapeutic drug screening study confirmed that 6-methoxyflavone has an inhibitory effect on HeLa cells [9]. Although an anticancer effect of 6-methoxyflavone on HeLa cells was previously observed, the specific mechanism of action has not been described.

During this study, we aimed to explore the potential role of 6-methoxyflavone in HeLa cells. First, microarray analyses, transcriptome sequencing analyses, functional enrichment analyses, and gene set enrichment analyses were performed to clarify the roles of 6-methoxyflavone in the cervical cancer cell cycle. Cell cycle assays further indicated that 6-methoxyflavone markedly induced S-phase arrest. Furthermore, transcriptome sequencing, polymerase chain reactions, western blotting, molecular docking, and noncovalent interaction analyses were used to clarify the mechanism of action of 6-methoxyflavone, and it was discovered that 6-methoxyflavone markedly induced S-phase arrest via the cyclin A2 (CCNA2)/cyclin-dependent kinase 2 (CDK2)/cyclin-dependent kinase inhibitor 1A (p21CIP1) pathway in HeLa cells.

## Materials and methods

There were no ethical issues involved in this study.

### Putative targets of 6-methoxyflavone

The three-dimensional conformer of 6-methoxyflavone was downloaded from the PubChem database [12]. The putative targets of 6-methoxyflavone were obtained from the PharmMapper database [^13–15^]. Only the human protein target set (version 2010) was selected. Finally, we identified 178 human protein targets of 6-methoxyflavone.

## Functional enrichment analysis

The 178 targets of 6-methoxyflavone were used for Gene Ontology (GO) and Kyoto Encyclopedia of Genes and Genomes (KEGG) analyses of the DAVID database (version 6.8) [16,17]. We used a threshold of p < 0.05 to filter the items downloaded from DAVID. Finally, we used GraphPad Prism 8.0.1 (https://www.graphpad.com/scientific-software/prism/), the GOplot package [18] of R 4.1.2 (https://www.r-project.org/), and the Jvenn online tool [19] to visualize the enrichment items of the targets.

### Microarray analysis

The raw data of mRNA expression and clinical characteristics of 304 cervical cancer samples and three healthy cervical samples were downloaded from The Cancer Genome Atlas database (http://cancergenome.nih.gov/). We used Perl 5.30.3 (https://www.perl.org/) to identify mRNAs and lncRNAs. We used the R package edgeR [20] to export the mRNA expression profiles of cervical cancer cases and identified differentially expressed mRNAs (DEmRNAs) in cancer tissues and healthy tissues. The thresholds of |log_2_ fold change| and the adjusted p value were set as 1.0 and 0.05, respectively. GraphPad Prism was used to statistically analyze clinical data. The Jvenn online database was used to visualize the intersection between the targets and DEmRNAs.

### Gene set enrichment analysis

A gene set enrichment analysis was performed to explore the functions of intersecting genes in cervical cancer. First, we obtained the expression data of the intersecting genes identified in the mRNA expression profile of cervical cancer. Then, we extracted the expression data of the intersecting genes from the profiles. We divided the intersecting genes into high-expression and low-expression groups using the median method and generated a phenotype data file. Finally, the two files were evaluated using gene set enrichment analysis version 4.0.3 [21,22], the website’s gene matrix, and signal2noise method. Significant terms with q < 0.01 were selected. These terms were ranked within each dataset according to the normalized enrichment score. GraphPad Prism and the ggplot2 R package [23] were used to visualize the results.

### Transcriptome sequencing

After HeLa cells were cultured with 0.16% dimethyl sulfoxide (DMSO) and 65 μM 6-methoxyflavone for 48 hours, the total RNA extraction reagent (Takara, Dalian, China) was applied to lyse the cells on ice [24]. RNA libraries were constructed, and transcriptome sequencing was performed using an Illumina Hiseq™ platform (Illumina Inc., San Diego, CA, USA) at Sangon Biotech Co., Ltd. (Shanghai, China). High-throughput sequencing data quality control and genomic contamination were assessed using FastQC version 0.11.2 software (http://www.bioinformatics.babraham.ac.uk/projects/fastqc/). Differentially expressed transcripts were identified as ≥5 transcripts per million, an absolute value of log2 fold change >1, and q < 0.05. Pathways and biological process enrichment analyses were performed and visualized using the KEGG, DAVID databases, and GraphPad Prism version 8.0.1 for Windows.

### Small molecule compound and cell culture

6-Methoxyflavone (CAS: 26,964–24-9) was obtained from Weikeqi Biological Technology Co., Ltd. (Chengdu, China). The compound powder was dissolved in DMSO under ultrasonic oscillation. Human cervical cancer cell lines (HeLa, C33A, and SiHa) and a human immortal keratinocyte line (HaCaT) were obtained from the Institute of Basic Medical Sciences of the Chinese Academy of Medical Sciences (Beijing, China). The cells were seeded in treated tissue culture plates or flasks (25 cm^2^) in modified Eagle’s medium or Dulbecco’s modified Eagle’s medium (HyClone, Logan, UT, USA) supplemented with 10% fetal bovine serum. The temperature and carbon dioxide concentration of the incubator were set at 37°C and 5%, respectively [25].

## Cell proliferation assessment

The cell counting kit-8 (CCK-8, Beyotime, Shanghai, China) was used to measure the inhibition rates of HaCaT, HeLa, C33A, and SiHa cell proliferation [26]. Single-cell suspensions were resuspended at a concentration of 6 × 10^4^ cells/mL after centrifugation (1000 rpm for 5 min). Single-cell suspensions (100 μL) were seeded in each well of a 96-well plate and incubated for 24 hours. The experimental design was as follows: six complexes per concentration, six concentration levels (0.16% DMSO, 20 μM, 40 μM, 80 μM, 120 μM, and 160 μM), and three test durations (24** **hours, 48 hours, and 72 hours). GraphPad Prism was used to analyze the microplate reader (Thermo Fisher Scientific™, Madison, WI, USA) optical density values and to calculate the half maximal inhibitory concentration (IC50) and cell inhibition rates.

### Cell cycle assessment

HeLa cells (1.2 × 10^6^) were equally seeded in four wells of a six-well plate and incubated for 24 hours. Four concentrations (0.16% DMSO, 20 μM, 80 μM, and 160 μM) of 6-methoxyflavone were added to the HeLa cells and incubated for an additional 48 hours. A DNA content quantitation assay (Solarbio, Beijing, China) was used to detect the cell cycle. According to the instructions, 100 μL of RNase A solution and 400 μL of propidium iodide were added to each loading tube and incubated for 20 minutes at 4°C. A flow cytometer (Beckman Coulter, Fullerton, CA, USA) was used to record the fluorescence intensity at 488 nm. FlowJo 10.5.3 (TreeStar, Ashland, CA, USA) was used to calculate the fitting curve, and GraphPad Prism was used to analyze the percentage of HeLa cells in the different cell cycle phases [27].

### Quantitative polymerase chain reaction

HeLa cells (8× 10^5^) were seeded in two wells of a six-well plate and incubated for 24 hours. Two concentrations (0.16% DMSO and 65 μM) of 6-methoxyflavone were added, and the cells were incubated for an additional 48 hours. RNAiso plus reagent (Takara, Dalian, China) was added to each treated sample to extract total RNA. The PrimeScript™ RT and gDNA Eraser kit (Takara) was used to erase genomic DNA and synthesize complementary DNA. The TB Green® Premix Ex Taq™ II kit (Takara) and StepOnePlus real-time quantitative polymerase chain reaction (PCR) system (Thermo Fisher Scientific, Madison, WI, USA) were used to perform PCR. Glyceraldehyde 3-phosphate dehydrogenase (Sangon Biotech, Shanghai, China) was used as the internal reference. Finally, relative mRNA expression was calculated using the Livak method [24]. Primer sequences are listed in Table 1.

**Table 1.** Primer sequences used for the quantitative polymerase chain reaction

Primer (human)	Forward	Reverse
CCNA2	CGCTGGCGGTACTGAAGTC	GAGGAACGGTGACATGCTCAT
CCNB1	AATAAGGCGAAGATCAACATGGC	TTTGTTACCAATGTCCCCAAGAG
CCND1	GCTGCGAAGTGGAAACCATC	CCTCCTTCTGCACACATTTGAA
CCNE1	AAGGAGCGGGACACCATGA	ACGGTCACGTTTGCCTTCC
CDK1	AAACTACAGGTCAAGTGGTAGCC	TCCTGCATAAGCACATCCTGA
CDK2	CCAGGAGTTACTTCTATGCCTGA	TTCATCCAGGGGAGGTACAAC
CDK4	ATGGCTACCTCTCGATATGAGC	CATTGGGGACTCTCACACTCT
CDK6	GCTGACCAGCAGTACGAATG	GCACACATCAAACAACCTGACC
P16INK4A	GATCCAGGTGGGTAGAAGGTC	CCCCTGCAAACTTCGTCCT
p21CIP1	TGTCCGTCAGAACCCATGC	AAAGTCGAAGTTCCATCGCTC
TP53	CAGCACATGACGGAGGTTGT	TCATCCAAATACTCCACACGC
RB	CTCTCGTCAGGCTTGAGTTTG	GACATCTCATCTAGGTCAACTGC

Abbreviations: CCNA2: cyclin A2; CCNB1: cyclin B1; CCND1: cyclin D1; CCNE1: cyclin E1; CDK1: cyclin-dependent kinase 1; CDK2: cyclin-dependent kinase 2; CDK4: cyclin-dependent kinase 4; CDK6: cyclin dependent kinase 6; P16INK4: cyclin dependent kinase inhibitor 2A; p21CIP1: cyclin-dependent kinase inhibitor 1A; TP53: tumor protein p53; RB: retinoblastoma transcriptional corepressor 1.

### Western blot analysis

The HeLa cultivation and processing methods were the same as those used for PCR. Cell lysis buffer for western blotting and immunoprecipitation (Beyotime) and phenylmethanesulfonyl fluoride solution (Beyotime) were used to extract the protein samples. An enhanced bicinchoninic acid kit (Beyotime) was used to measure the protein concentration. Precontrast gels (4%-20%) (Beyotime) and polyvinylidene fluoride membranes (Merck Millipore, Burlington, VT, USA) were used for protein electrophoresis and transfer. The incubation times for 5% bovine albumin V, primary antibody, and secondary antibody were 1 hour, overnight, and 1.5 hours, respectively. The primary antibodies used were CCNA2, cyclin D1 (CCND1), cyclin E1 (CCNE1), cyclin dependent kinase 6 (CDK6), p21CIP1 rabbit polyclonal antibody, CDK2 rabbit monoclonal antibody (1:1000, Beyotime) and glyceraldehyde 3-phosphate dehydrogenase rabbit polyclonal antibody (1:5000, Sangon Biotech). The Amersham Imager 680 system (GE Healthcare, Little Chalfont, UK) and a chemiluminescence kit (Beyotime) were used to generate grayscale images of the protein bands [28].

### Affinity and noncovalent interactions between 6-methoxyflavone and receptors

The three-dimensional structure of 6-methoxyflavone was obtained from PubChem [12]. The crystal structures of CCNA2, CDK2, and p21CIP1 were obtained from the AlphaFold [29,30] database. The crystal structure of the complex between CCNA2 and CDK2 (2BKZ) [31] was downloaded from the Protein Data Bank (https://www.rcsb.org/). AutoDockTools (version 1.5.6) (http://autodock.scripps.edu/) was used to generate pdbqt format files, add hydrogen, and determine the active site. AutoDock Vina [32] was used to perform molecular docking and determine the binding free energy. The minimum free energy of each compound–receptor complex was converted to the inhibition constant (Ki)/dissociation constant (Kd). The ideal gas constant was 1.986 cal/K and the room temperature was 298 K (25°C).

PyMOL 2.2 (https://pymol.org/2/) was used to visualize the docking results. The PDB format files of the 6-methoxyflavone–receptor complexes were submitted to the Protein–Ligand Interaction Profiler [33] database. The noncovalent interactions and binding sites between 6-methoxyflavone and receptors were analyzed using the identifier function of the Protein–Ligand Interaction Profiler database.

### Analyses of clinical characteristics

The drug sensitivity data of CCNA2, CCND1, CCNE1, CDK2, CDK2, and p21CIP1 were downloaded from the RNAactDrug database (http://bio-bigdata.hrbmu.edu.cn/RNAactDrug/

index.jsp). The data of six target expression levels and clinical characteristics of 304 cervical cancer samples were downloaded from The Cancer Genome Atlas. We divided the six targets into high-expression and low-expression groups according to the median. Disease free survival data was obtained from Gene Expression Profiling Interactive Analysis database [34]. GraphPad Prism software and Jvenn database were used to analyze and visualize the results.

### Statistical analysis

GraphPad Prism was used for the statistical analysis. All experimental operations were performed for three biological replicates. All results were analyzed by a paired t test, one-way analysis of variance (ANOVA), or chi-square test. A nonlinear regression analysis was performed to calculate the IC50, and the log-rank test was performed for the survival analysis. Significant differences were indicated by p < 0.05.

## Results

This study aimed to explore the molecular mechanisms of 6-methoxyflavone in HeLa cells. First, microarray analyses, transcriptome sequencing analyses, functional enrichment analyses, and gene set enrichment analyses were used to clarify the roles of 6-methoxyflavone in cervical cancer. Cell cycle assays, transcriptome sequencing, polymerase chain reactions, western blotting, molecular docking, and noncovalent interaction analyses were used to determine the molecular mechanism of 6-methoxyflavone in HeLa cells.

### Functional enrichment analysis and hub gene identification

**Figure 1.**
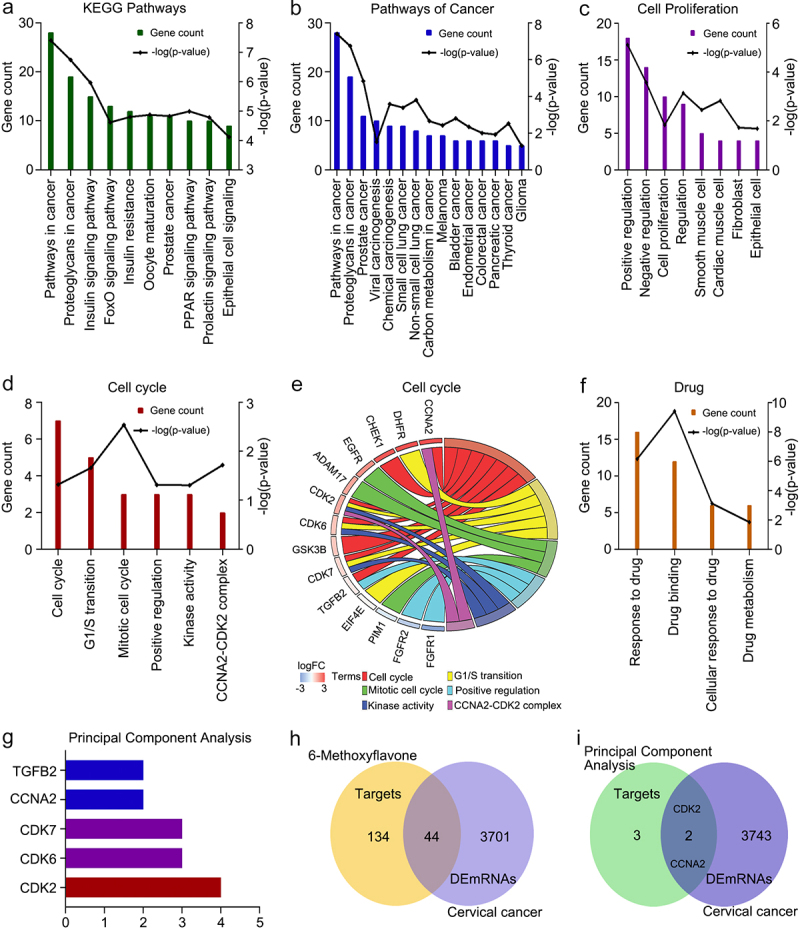
Functional enrichment analyses of 178 targets. **A**. The 10 most common KEGG pathways (p < 0.0001). **B**. KEGG pathways related to cancer. **C**. Biological processes related to cell proliferation. **D and F**. Functional enrichment terms related to cell cycles and drugs (p < 0.05). **E**. Genes enriched in six cell cycle items. **G**. Principal component analysis of the genes enriched in the cell cycle. **H**. Intersections of the 178 targets and the DEmRNAs of cervical cancer. **I**. Intersections among the targets of the principal component analysis and DEmRNAs. Abbreviations: KEGG: Kyoto Encyclopedia of Genes and Genomes; DEmRNAs: differentially expressed mRNAs; FoxO: forkhead box O; PPAR: peroxisome proliferator activated receptor; CCNA2: cyclin A2; DHFR: dihydrofolate reductase; CHEK1: checkpoint kinase 1; EGFR: epidermal growth factor receptor; ADAM17: ADAM metallopeptidase domain 17; CDK2: cyclin-dependent kinase 2; CDK6: cyclin dependent kinase 6; CDK7: cyclin-dependent kinase 7; GSK3B: glycogen synthase kinase 3 beta; TGFB2: transforming growth factor beta 2; EIF4E: eukaryotic translation initiation factor 4E; PIM1: Pim-1 proto-oncogene, serine/threonine kinase; FGFR1: fibroblast growth factor receptor 1; FGFR2: fibroblast growth factor receptor 2.

We obtained 178 targets of 6-methoxyflavone from the PharmMapper database. We identified 56 KEGG pathways, 200 biological processes, 32 cellular components, and 89 molecular functions in DAVID. Figure 1a shows the 10 most common KEGG pathway terms (p < 0.0001). 6-Methoxyflavone was mainly correlated with cancer-related pathways. The GO and KEGG enrichment analyses indicated that the targets were related to 15 KEGG cancer pathways (Figure 1b), eight cell proliferation biological processes (Figure 1c), six cell cycle terms (Figure 1d), and four drug terms (figure 1f) (p < 0.05). Figure 1e shows that the cell cycle terms were significantly enriched with 14 genes (p < 0.05). A principal component analysis of the 14 genes showed that 6-methoxyflavone was closely associated with five genes. The most important factor was CDK2 expression (Figure 1g).

We analyzed the mRNA expression data of 304 cervical cancer tissues and three healthy tissues. A total of 3745 mRNAs were identified as DEmRNAs. The intersections among the 178 targets and 3745 DEmRNAs are shown in Figure 1h. Of the 178 targets, 24.7% were cervical cancer DEmRNAs. Two genes (CCNA2 and CDK2) showed overlaps between the principal component analysis data and DEmRNA data (Figure 1i). CCNA2 and CDK2 are highly expressed in cervical cancer.

### Gene set enrichment analysis

**Figure 2.**
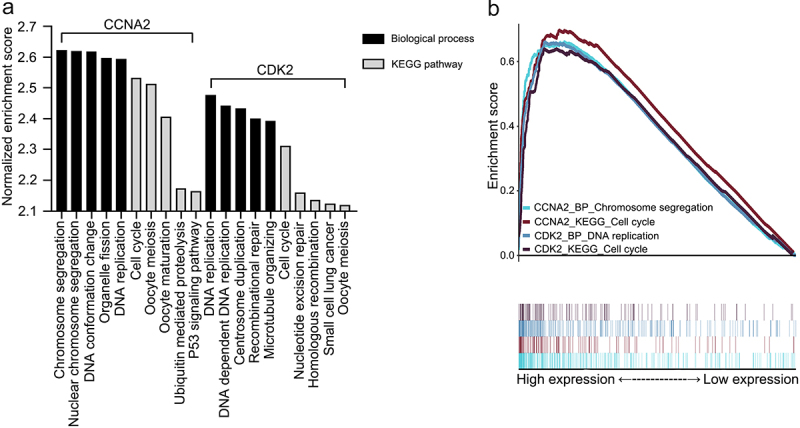
Gene set enrichment analyses of CCNA2 and CDK2. **A**. The five most common KEGG pathways or biological processes of each dataset (q < 0.01). **B**. The highest normalized enrichment score terms of each dataset. Abbreviations: KEGG: Kyoto Encyclopedia of Genes and Genomes; P53: tumor protein p53; CCNA2: cyclin A2; CDK2: cyclin-dependent kinase 2.

The 18,013 mRNA expression profiles of 304 cervical cancer samples were obtained from The Cancer Genome Atlas. Overall, 308 biological processes and 9 KEGG pathways were enriched in the high-CCNA2 expression group, whereas 153 biological processes and eight KEGG pathways were enriched in the high CDK2 expression group (q < 0.01). Figure 2a shows the five terms with the highest normalized enrichment scores for each dataset. Figure 2b shows that the highest normalized enrichment score terms in these four datasets were chromosome segregation, cell cycle, DNA replication, and cell cycle. It is unclear whether chromosome segregation and DNA replication are involved in cell cycle progression. Enrichment scores were greater than zero. The results indicated that high CCNA2 expression and high CDK2 expression promoted cell cycle progression in cervical cancer.

### 6-Methoxyflavone was significantly related to the cell cycle or proliferation

**Figure 3.**
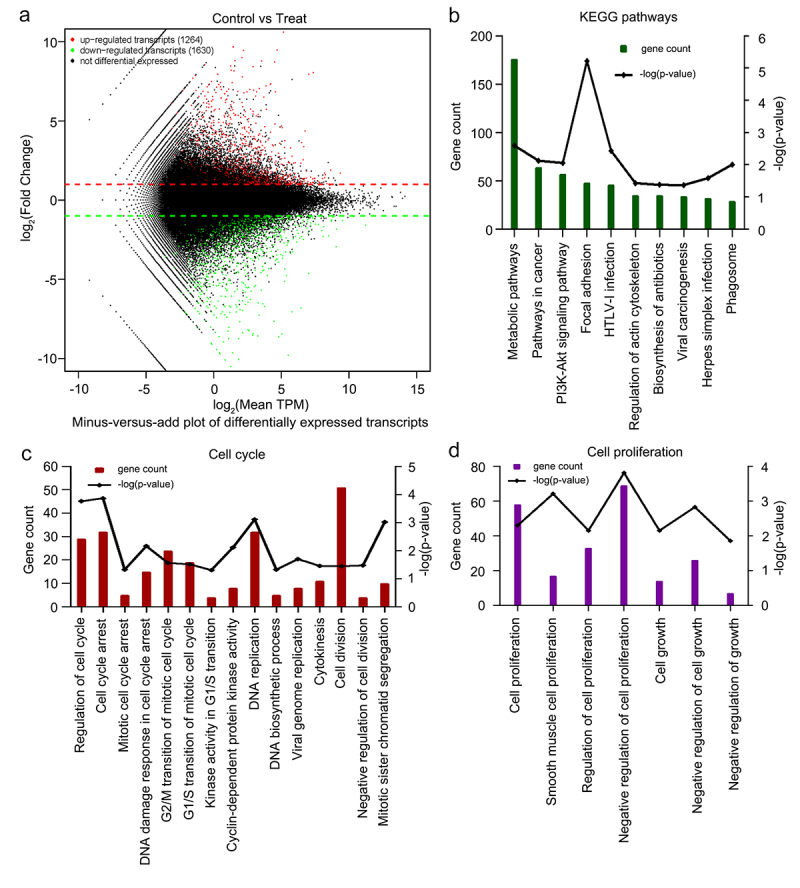
Eukaryotic transcriptome sequencing results of HeLa cells after 6-methoxyflavonoid treatment. Three control samples were treated with 0.16% dimethyl sulfoxide. Three treatment samples were treated with 65 μM 6-methoxyflavone. A. Minus versus add plot of differential expressed transcripts. B. The 10 most common KEGG pathways of differential expressed transcripts (p < 0.05). C and D. Gene oncology enrichment results of differential expressed transcripts. Cell cycle-related or proliferation-related biological processes (p < 0.05). Abbreviations: TPM: transcripts per million; HTLV-I: human T-lymphotropic virus type I; PI3K: phosphoinositide 3-kinase; Akt: protein kinase B.

To elucidate the anticancer effects of 6-methoxyflavone, we used eukaryotic transcriptome sequencing to identify significantly enriched biological processes and KEGG pathways in human HeLa cells treated with 6-methoxyflavone. High-throughput sequencing data quality control results showed that all six samples were of acceptable quality and were free from contamination (**Supplement** Tables S1 **and** S2). Compared with the three 0.16% DMSO-treated control samples, a total of 2894 significantly differentially expressed transcripts in the three samples treated with 65 μM 6-methoxyflavone were observed. Among these 2894 transcripts, 1264 were upregulated and 1630 were downregulated (Figure 3a). Figure 3b shows the 10 most common KEGG pathways significantly enriched by 6-methoxyflavonoid (p < 0.05). 6-Methoxyflavonoid was significantly associated with pathways in cancer and viral carcinogenesis. We obtained 181 significantly enriched biological processes using GO analysis. 6-Methoxyflavone was significantly related to the 15 cell cycle biological processes (Figure 3c) and seven cell proliferation (Figure 3d) biological processes (p < 0.05).

### 6-Methoxyflavone inhibited cell proliferation

**Figure 4.**
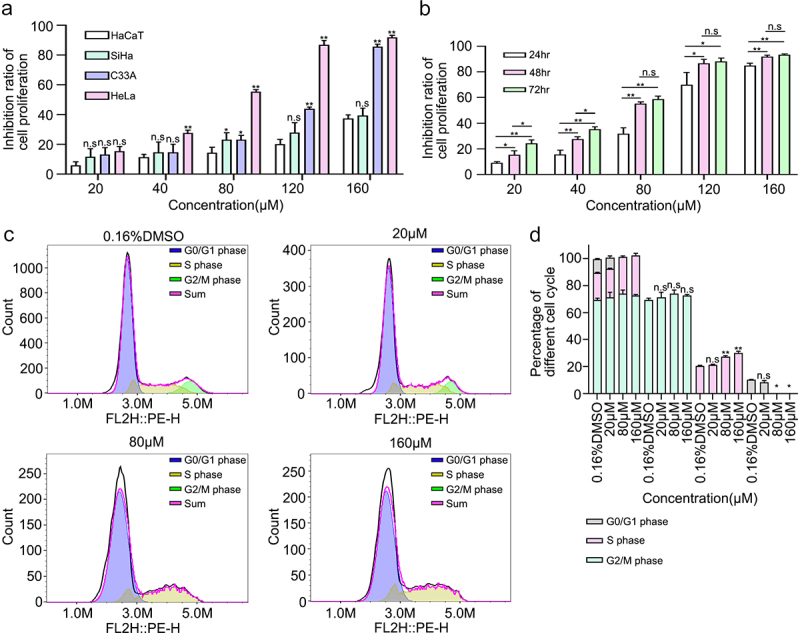
6-Methoxyflavone inhibits cervical cancer cell proliferation and induces S-phase arrest in HeLa cells. **A**. Inhibition ratios of HaCaT, HeLa, C33A, and SiHa cells. The four cells were treated with six concentrations of 6-methoxyflavone (0.16% DMSO, 20 μM, 40 μM, 80 μM, 120 μM, and 160 μM) for 48 hours. Six replicate holes were used at each concentration level. A water-soluble tetrazolium salt-8 kit was used to measure the inhibitory effects. **B**. Inhibition ratio of HeLa cell proliferation. HeLa cells were treated with six concentrations of 6-methoxyflavone for 24 hours, 48 hours, and 72 hours. **C**. After propidium iodide staining, the distribution of HeLa cell cycle phases was measured using flow cytometry. **D**. The histogram shows the ratio of the HeLa cell cycle phases in Figure 3c. All experiments were performed using three biological replicates. Statistical analysis was performed using a one-way analysis of variance (ANOVA). *p < 0.05. **p < 0.01. n.s.: not significant. DMSO: dimethyl sulfoxide.

A functional enrichment analysis of the 178 targets showed that 6-methoxyflavone was closely related to pathways related to cancer and cell proliferation. Therefore, a CCK-8 kit was used to detect the inhibitory effects of 6-methoxyflavone on cervical cancer cells. First, we treated HaCaT, C33A, SiHa, and HeLa cells at six concentrations (0.16% DMSO, 20 μM, 40 μM, 80 μM, 120 μM, and 160 μM) of 6-methoxyflavone for 48 hours. We compared C33A, SiHa, and HeLa cell inhibition with HaCaT cell inhibition and found that the difference between HeLa and HaCaT cells was the most significant (Figure 4a). HeLa cells were most sensitive to 6-methoxyflavone, which is consistent with the results of a previous study [9]. Next, we treated HeLa cells with six concentrations of 6-methoxyflavone for 24 hours, 48 hours, and 72 hours. The IC50 values of 24 hours, 48 hours, and 72 hours were 94.05 μM, 62.24 μM, and 52.12 μM, respectively. Compared with 24 hours, the inhibition rates at 48 hours and 72 hours were significantly higher. However, there were no significant differences between 48 hours and 72 hours at concentrations of 80 μM, 120 μM, and 160 μM (Figure 4b). Therefore, we treated HeLa cells with 65 μM 6-methoxyflavone for 48 hours during subsequent PCR and western blot analyses.

### 6-Methoxyflavone induced HeLa cell S-phase arrest

We detected the percentages of the different cell cycle phases and found that as the concentration of 6-methoxyflavone increased, the percentage of S-phase cells increased and the G2/M phase was reversed. 6-Methoxyflavone significantly increased the percentage of S-phase cells in a concentration-dependent manner. These findings indicate that 6-methoxyflavone significantly induced S-phase arrest in HeLa cells (Figure 4c, D).

Because the results of the cell cycle assessment showed that 6-methoxyflavone induced S-phase arrest in HeLa cells, we next explored whether 6-methoxyflavone affected the relative mRNA and protein expression levels in the cell cycle pathway.

**Figure 5.**
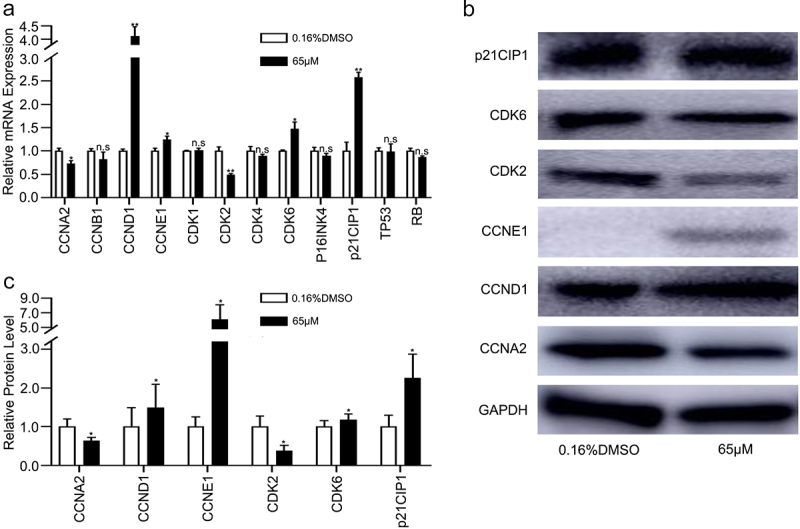
6-Methoxyflavone induces S-phase arrest through the CCNA2/CDK2/p21CIP1 pathway. HeLa cells were treated with 6-methoxyflavone (0.16% DMSO and 65 μM) for 48 hours. Cell cycle-related mRNA and protein expression were measured by PCR and western blotting. A. The relative mRNA expressions of CCNA2, CCNB1, CCND1, CCNE1, CDK1, CDK2, CDK4, CDK6, P16INK4, p21CIP1, TP53, and RB. B and C. Protein expressions of CCNA2, CCND1, CCNE1, CDK2, CDK6, and p21CIP1. Each assay was performed in triplicate. Statistical analysis was performed using the paired t-test. *p < 0.05. **p < 0.01. n.s.: not significant. Abbreviations: CCNA2: cyclin A2; CCNB1: cyclin B1; CCND1: cyclin D1; CCNE1: cyclin E1; CDK1: cyclin-dependent kinase 1; CDK2: cyclin-dependent kinase 2; CDK4: cyclin-dependent kinase 4; CDK6: cyclin dependent kinase 6; P16INK4: cyclin dependent kinase inhibitor 2A; p21CIP1: cyclin-dependent kinase inhibitor 1A; TP53: tumor protein p53; RB: retinoblastoma transcriptional corepressor 1.

The relative mRNA and protein expression levels of CCNA2 and CDK2 in the 6-methoxyflavone treatment group (65 μM) were significantly lower than those of the control group (0.16% DMSO), however, the mRNA and protein expression levels of CCND1, CCNE1, CDK6, and p21CIP1 were significantly higher (Figure 5). However, mRNA expression levels of cyclin B1 (CCNB1), cyclin-dependent kinase 1 (CDK1), cyclin-dependent kinase 4 (CDK4), cyclin dependent kinase inhibitor 2A (P16INK4), tumor protein p53 (TP53), and retinoblastoma transcriptional corepressor 1 (RB) were not significantly affected by 6-methoxyflavone (Figure 5a). The PCR and western blot results indicated that 6-methoxyflavone induced S-phase arrest via the cyclin A2 (CCNA2)/CDK2/p21CIP1 pathway. Each phase of the cell cycle contributed to the entire cycle. The mRNA expression levels of CCND1, CCNE1 and CDK6 were affected by 6-methoxyflavone. These mRNA expression levels were consistent with the results of transcriptome sequencing (Table 2).

**Table 2.** Core mRNA biomarker expression levels of transcriptome sequencing

GeneName	GeneID	Transcript id	log2FoldChange	qValue
CCNA2	ENSG00000145386	ENST00000618014	−0.8277	0.0003
CCND1	ENSG00000110092	ENST00000227507	0.8394	2.66E-11
CCNE1	ENSG00000105173	ENST00000357943	0.6317	0.8789
CDK2	ENSG00000123374	ENST00000554619	−1.3566	0.0358
CDK6	ENSG00000105810	ENST00000265734	1.0095	0.0247
p21CIP1	ENSG00000124762	ENST00000615513	1.2123	0.0058

Abbreviations: CCNA2: cyclin A2; CCND1: cyclin D1; CCNE1: cyclin E1; CDK2: cyclin-dependent kinase 2; CDK6: cyclin dependent kinase 6; p21CIP1: cyclin-dependent kinase inhibitor 1A.

### Affinity and noncovalent interactions between 6-methoxyflavone and receptors

**Figure 6.**
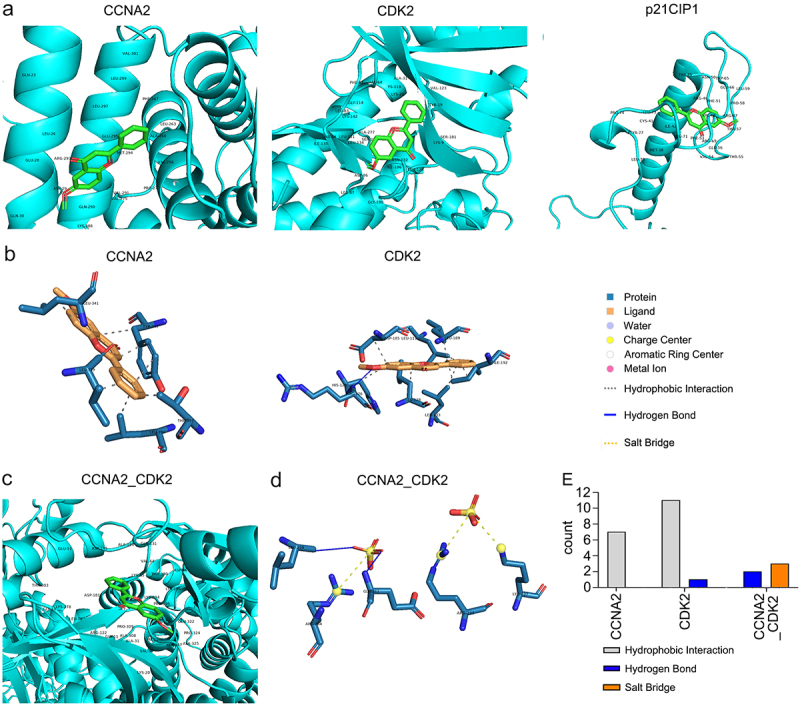
Affinity and noncovalent interactions between 6-methoxyflavone and receptors. **A and C**. The results of molecular dockings between 6-methoxyflavone and CCNA2, CDK2, p21CIP1, and the CCNA2–CDK2 complex. B and D. The noncovalent interactions between 6-methoxyflavone and CCNA2, CDK2, and CCNA2–CDK2 complex. **E**. The noncovalent interactions between 6-methoxyflavone and CCNA2, CDK2, and the CCNA2–CDK2 complex. Abbreviations: CCNA2: cyclin A2; CDK2: cyclin-dependent kinase 2; p21CIP1: cyclin-dependent kinase inhibitor 1A.

The minimum free energy levels between 6-methoxyflavone and CCNA2, CDK2, p21CIP1, and the CCNA2–CDK2 complex were −7.1 kcal/M, −8.6 kcal/M, −6.2 kcal/M, and −8.8 kcal/M, respectively (Figure 6a, C; Table 3). The corresponding Ki/Kd values were 6.16 μM, 0.49 μM, 28.2 μM, and 0.35 μM (Table 3). In this pathway, 6-methoxyflavone had the strongest affinity toward and inhibitory effects on CDK2. The combination of CDK2 and CCNA2 enhanced these effects. The noncovalent interactions between 6-methoxyflavone and the receptors consisted of seven (CCNA2) or 11 (CDK2) hydrophobic interactions, one (CDK2) or two (CCNA2–CDK2 complex) hydrogen bonds, and three salt bridges (CCNA2–CDK2 complex) (Figure 6b, D, E). In contrast, there were no noncovalent interactions between 6-methoxyflavone and p21CIP1.

**Table 3.** Molecular docking parameters of 6-methoxyflavone and receptors

Targets	Ligand	Interactions	Minimum binding affinity (kcal/M)	Ki/Kd values (μM)	Distance/Bond length (Å)
CCNA2	6MF	Hydrophobic Interactions	−7.1	6.16	2.49
CDK2	6MF	Hydrophobic Interactions	−8.6	0.49	2.30
CDK2	6MF	Hydrogen Bonds	−8.6	0.49	2.73
CCNA2_CDK2	6MF	Hydrogen Bonds	−8.8	0.35	3.625
CCNA2_CDK2	6MF	Salt Bridge	−8.8	0.35	4.20

Abbreviations: Ki: inhibition constant; Kd: dissociation constant; 6MF: 6-Methoxyflavone

### Clinical characteristic analysis

**Figure 7.**
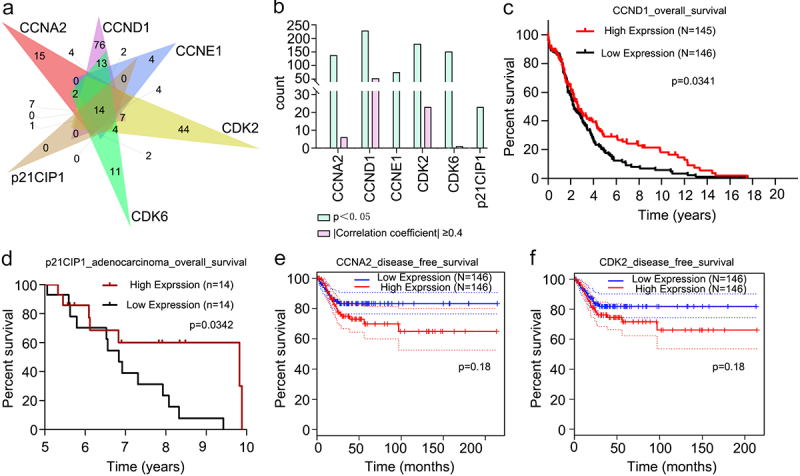
Clinical characteristics analyses of six receptors. **A**. Drug sensitivity data of CCNA2, CCND1, CCNE1, CDK2, CDK2, and p21CIP1. Numbers of drugs related to the six receptors (p < 0.05). **B**. Drugs related to the six receptors (p < 0.05). Drugs significantly related to the expressions of CCNA2, CCND1, CDK2, and CDK6 (p < 0.05 and |Pearson and Spearman correlation coefficient|≥0.4). **C**. Overall survival analysis of cervical cancer patients in CCND1 high-expression and low-expression groups (p < 0.05). **D**. Overall survival analysis of cervical adenocarcinoma patients in p21CIP1 high-expression and low-expression groups(p < 0.05). E and F. Disease free survival analyses of cervical cancer patients in CCNA2 and CDK2 different expression groups.

Abbreviations: CCNA2: cyclin A2; CCND1: cyclin D1; CCNE1: cyclin E1; CDK2: cyclin-dependent kinase 2; CDK6: cyclin-dependent kinase 6; p21CIP1: cyclin-dependent kinase inhibitor 1A.

Functional enrichment analyses showed that the targets were related to the four drug terms (figure 1f) (p < 0.05). Therefore, we downloaded the drug sensitivity data of six receptors from RNAactDrug. CCNA2, CCND1, CCNE1, CDK2, CDK2, and p21CIP1 were related to 137, 228, 75, 179, 151, and 23 drugs, respectively (p < 0.05) (Figure 7a). Then, we performed a secondary screening of the data by applying p < 0.05. We set the filter condition as |Pearson and Spearman correlation coefficients|≥0.4. The expression levels of CCNA2, CCND1, CDK2 and CDK6 were closely associated with six drugs, 52 drugs, 23 drugs, and one drug, respectively (Figure 7a, B). 6-Methoxyflavone may regulate the sensitivity of other drugs. Survival analyses of the six receptors showed that the survival rates of the high CCND1 expression and low CCND1 expression groups were significantly different (p < 0.05) (Figure 7c). The five-year to 10-year overall survival rates of the high p21CIP1 expression and low p21CIP1 expression groups were significantly different (p < 0.05) (Figure 7d). Disease free survival rates in the low CCNA2 expression and low CDK2 expression groups were higher than corresponding high-expression groups (Figure 7e, F). The mRNA expression levels of CCND1, CCNE1, CDK2, CDK6, and p21CIP1 were significantly related to the histological classification of cervical cancer (p < 0.05). The mRNA expression levels of CDK2, CDK6, and p21CIP1 were significantly related to the age, primary tumor, and Federation International of Gynecology and Obstetrics stage of the patients (p < 0.05) (Table 4).

**Table 4.** Relationships between mRNA expression levels of six targets and the clinicopathological parameters of cervical cancer patients

Parameters	Relative expression of CCNA2			Relative expression of CCND1			Relative expression of CCNE1			Relative expression of CDK2			Relative expression of CDK6			Relative expression of p21CIP1		
	High	Low	P	High	Low	P	High	Low	P	High	Low	P	High	Low	P	High	Low	P
Age (n = 304)																		
≥60 years old	34	31	0.67	33	32	0.89	31	34	0.67	25	40	0.04*	27	38	0.12	33	32	0.89
<60 years old	118	121		119	120		121	118		127	112		125	114		119	120	
Histological classification (n = 304)																		
Squamous cell cancer	130	122	0.22	133	119	0.03*	133	119	0.03*	137	115	<0.01**	146	106	<0.01**	144	108	<0.01**
Adenocarcinoma	22	30		19	33		19	33		15	37		6	46		8	44	
Primary tumor (n = 241)																		
T1-T2	102	109	0.86	96	115	0.14	104	107	0.45	104	107	0.94	96	115	0.03*	104	107	0.15
T3-T4	15	15		18	12		17	13		15	15		20	10		19	11	
Lymph node invasion (n = 193)																		
N0	64	69	0.65	61	72	0.59	63	70	0.93	67	66	0.36	61	72	0.34	65	68	0.32
N1	31	29		30	30		28	32		26	34		32	28		34	26	
FIGO stage (n = 297)																		
Stage I–II	112	119	0.28	113	118	0.71	113	118	0.56	120	111	0.35	113	118	0.31	106	125	0.02*
Stage III–IV	37	29		34	32		35	31		30	36		37	29		41	25	

Statistical analyses were performed using the chi-square test. *p < 0.05, **p < 0.01. Abbreviations: FIGO: Federation International of Gynecology and Obstetrics

## Discussion

Natural products have become an important adjuvant therapy for cancer and are an effective way of preventing chemoresistance [35]. Cell and animal experiments indicated that glycyrrhizic acid, quercetin, and homoharringtonine exhibited strong anticancer activity in colon and colorectal cancer progression by inhibiting sirtuin 3, phosphoinositide 3-kinase (PI3K)/protein kinase B (AKT), and PI3K/AKT/mechanistic target of rapamycin kinase (mTOR) signaling pathways [^36–38^].

In vivo and in vitro experiments related to breast cancer have shown that Imperatae rhizoma extract inhibited cell viability and tumor growth [39]. 6-Methoxyflavone is an important active ingredient in Imperatae rhizoma. A HeLa cell proliferation assessment also indicated that 6-methoxyflavone has anticancer activity [9]. However, the specific anticancer mechanism of 6-methoxyflavone has not yet been elucidated. During this study, we identified 178 putative targets of 6-methoxyflavone. Functional enrichment analyses of the targets and transcriptome sequencing results indicated that 6-methoxyflavone was significantly related to cancer, cell proliferation, and the cell cycle (p < 0.05). We identified intersections between two highly expressed genes (CCNA2 and CDK2) for five cell cycle principal component analysis targets and 3745 DEmRNAs. The gene set enrichment analysis results of CCNA2 and CDK2 indicated that high mRNA expressions of CCNA2 and CDK2 stimulate cell cycle progression in cervical cancer. Compared with a series of studies of colon cancer and colorectal cancer, this study also evaluated molecular docking and noncovalent interactions between 6-methoxyflavone and receptors.

Cyclins and cyclin-dependent kinases have an important role in cell cycle regulation and cell proliferation. Overexpression of Roundabout homolog 1 caused S-phase arrest to inhibit pancreatic cancer cell proliferation by significantly reducing the expressions of CCNA2 and CDK2 [40]. Cyclin B2 (CCNB2) knockdown significantly suppressed cell proliferation and induced G2/M-phase arrest in acute myeloid leukemia cells [41]. Suppression of cell division cycle-associated 3 induced G0/G1-phase arrest to inhibit prostate cancer cell proliferation via cyclin D1 signaling inactivation and p21CIP1 accumulation [42]. Silencing of tumor susceptibility 101 reduced proliferation and induced G0/G1 arrest by markedly decreasing the expression of cyclin E1/CDK2 in renal cell carcinoma [43]. The S phase is an important process in DNA replication and synthesis during the cell cycle. S-phase arrest results in failures in DNA replication, mitosis [44], cell growth, and proliferation [^45–47^]. The cyclin/cyclin-dependent kinase (CDK)/CDK inhibitor (CKI) network is the core regulatory pathway of the cell cycle. The CCNA1/CCNB1/CDK1/p21CIP1 pathway is associated with G2/ M-phase arrest in glioblastoma cells [48]. The CCNB/CDK/cyclin dependent kinase inhibitor 1B pathway was confirmed to be related to cell cycle progression in prostate cancer [49]. The cyclin/CDK1/CKI pathway was found to affect cell cycle distribution and proliferation of human oral squamous cell carcinoma cells [50]. During this study, we found that 6-methoxyflavone inhibited proliferation and induced S-phase arrest through the CCNA2/CDK2/p21CIP1 signaling pathway in HeLa cells.

The purpose of all basic medical experimental research is to improve the clinical status of patients. The clinical characteristic analysis results showed that 6-methoxyflavone might be related to clinicopathological parameters (drug sensitivity, survival rate, histological classification, age, primary tumor, and Federation International of Gynecology and Obstetrics stage stage) of cervical cancer patients. Therefore, 6-methoxyflavone could be a possible treatment for cervical cancer.

## Conclusion

During this study, we explored the effect of 6-methoxyflavone on HaCaT, SiHa, C33A, and HeLa cells using a CCK-8 kit and a cell cycle assay. The results revealed that 6-methoxyflavone inhibited HaCaT, SiHa, C33A, and HeLa cell proliferation and induced S-phase arrest in HeLa cells. Moreover, PCR and western blot results showed that 6-methoxyflavone induced S-phase arrest via the CCNA2/CDK2/p21CIP1 pathway. The CCNA2/CDK2/p21CIP1 pathway is the most critical molecular factor in S-phase regulation. The molecular docking and noncovalent interaction analysis results showed that 6-methoxyflavone had the strongest affinity toward, inhibitory effect on, and noncovalent interactions with CDK2, and the combination of CDK2 and CCNA2 enhanced these effects.

The clinical characteristic analyses showed that 6-methoxyflavone might be related to six clinicopathological parameters of cervical cancer patients. 6-Methoxyflavone could be a possible treatment for cervical cancer.

## Supplementary Material

Supplemental Material
