# Supplementary material for: 6-Methoxyflavone induces S-phase arrest through the CCNA2/CDK2/p21CIP1 signaling pathway in HeLa cells
Source: Bioengineered. 2022 Mar 4;13(3):7277–92. doi: 10.1080/21655979.2022.2047496 (PMC8973872; doi:10.1080/21655979.2022.2047496)
Supplement: Supplemental Material [file KBIE_A_2047496_SM2444.docx]

**Supplement table 1. Quality control data for six transcriptome sequencing samples**

| Terms | Treat1 | Treat2 | Treat3 | Control1 | Control2 | Control3 |
| --- | --- | --- | --- | --- | --- | --- |
| Total Reads Count(#) | 71683414 | 44367242 | 61212528 | 62908496 | 59908906 | 55318348 |
| Total Bases Count(bp) | 1.02E+10 | 6.3E+09 | 8.69E+09 | 8.9E+09 | 8.51E+09 | 7.88E+09 |
| Average Read Length(bp) | 142.9 | 141.93 | 141.92 | 141.47 | 142 | 142.43 |
| Q10 Bases Count(bp) | 1.02E+10 | 6.3E+09 | 8.69E+09 | 8.9E+09 | 8.51E+09 | 7.88E+09 |
| Q10 Bases Ratio(%) | 100.00% | 100.00% | 100.00% | 100.00% | 100.00% | 100.00% |
| Q20 Bases Count(bp) | 1E+10 | 6.14E+09 | 8.48E+09 | 8.7E+09 | 8.3E+09 | 7.68E+09 |
| Q20 Bases Ratio(%) | 97.66% | 97.55% | 97.60% | 97.72% | 97.55% | 97.48% |
| Q30 Bases Count(bp) | 9.53E+09 | 5.84E+09 | 8.07E+09 | 8.3E+09 | 7.89E+09 | 7.29E+09 |
| Q30 Bases Ratio(%) | 93.03% | 92.77% | 92.88% | 93.22% | 92.74% | 92.56% |
| N Bases Count(bp) | 222262 | 132377 | 185853 | 192849 | 184158 | 171865 |
| N Bases Ratio(%) | 0.00% | 0.00% | 0.00% | 0.00% | 0.00% | 0.00% |
| GC Bases Count(bp) | 5.36E+09 | 3.08E+09 | 4.51E+09 | 4.59E+09 | 4.41E+09 | 4.07E+09 |
| GC Bases Ratio(%) | 52.32% | 48.94% | 51.86% | 51.61% | 51.86% | 51.71% |

**Supplement table 2.** **Species distribution detection results of six transcriptome sequencing samples**

| Species | Treat1 | Treat2 | Treat3 | Control1 | Control2 | Control3 |
| --- | --- | --- | --- | --- | --- | --- |
| Homo sapiens | 4072 | 4357 | 4082 | 4231 | 4221 | 4124 |
| Pan paniscus | 4058 | 3864 | 4065 | 3869 | 3908 | 3991 |
| Ailuropoda melanoleuca | 546 | 465 | 526 | 502 | 515 | 559 |
| Gorilla gorilla | 380 | 412 | 410 | 417 | 349 | 386 |
| Eukaryotic synthetic | 227 | 235 | 248 | 320 | 306 | 268 |
| Hylobates moloch | 177 | 147 | 161 | 149 | 157 | 148 |
| Trachypithecus francoisi | 99 | 89 | 90 | 76 | 107 | 105 |
| Gateway entry | 41 | 41 | 39 | 40 | 32 | 40 |
| Nomascus leucogenys | 27 | 26 | 22 | 19 | 29 | 19 |
| Pan troglodytes | 27 | 34 | 36 | 44 | 36 | 37 |
| Chelonoidis abingdonii | 22 | 23 | 34 | 36 | 27 | 29 |
| Mirounga leonina | 21 | 33 | 22 | 30 | 28 | 24 |
| Human papillomavirus | 16 | 21 | 12 | 26 | 21 | 23 |
| Sapajus apella | 15 | 14 | 11 | 11 | 15 | 9 |
| Papio anubis | 14 | 21 | 15 | 14 | 20 | 10 |
| Aspergillus flavus | 12 | 5 | 8 | 3 | 7 | 6 |
| Rhinopithecus roxellana | 11 | 2 | 5 | 6 | 7 | 4 |
| Felis catus | 9 | 10 | 13 | 2 | 2 | 4 |
| Pongo abelii | 7 | 5 | 7 | 13 | 4 | 4 |
| Piliocolobus tephrosceles | 7 | 12 | 13 | 9 | 10 | 10 |
| Uncultured bacterium | 7 | 0 | 0 | 0 | 0 | 0 |
| Sturnira hondurensis | 6 | 3 | 1 | 9 | 6 | 9 |
| Aspergillus parasiticus | 4 | 0 | 6 | 1 | 1 | 0 |
| Human cytoskeletal | 3 | 1 | 5 | 3 | 1 | 2 |
| Acidibrevibacterium fodinaquatile | 2 | 0 | 0 | 0 | 0 | 0 |
| Sus scrofa | 2 | 0 | 0 | 0 | 1 | 0 |
| Marmota flaviventris | 2 | 2 | 2 | 2 | 1 | 1 |
| Danio rerio | 2 | 0 | 2 | 1 | 1 | 1 |
| Human ribosomal | 2 | 1 | 4 | 2 | 2 | 2 |
| Macaca mulatta | 2 | 3 | 6 | 1 | 4 | 1 |
| Expression vector | 2 | 0 | 1 | 1 | 0 | 2 |
| Phanerochaete pseudomagnoliae | 2 | 1 | 0 | 1 | 0 | 2 |
| Acipenser ruthenus | 2 | 1 | 6 | 1 | 2 | 0 |
| Condylura cristata | 1 | 0 | 0 | 0 | 0 | 0 |
| Erinaceus europaeus | 1 | 0 | 0 | 0 | 0 | 0 |
| Propithecus coquereli | 1 | 0 | 0 | 0 | 0 | 0 |
| Phyllostomus discolor | 1 | 0 | 0 | 0 | 0 | 0 |
| Sphingomonas lutea | 1 | 0 | 0 | 0 | 0 | 0 |
| Deinococcus proteolyticus | 1 | 0 | 0 | 0 | 0 | 0 |
| Paracoccus sanguinis | 1 | 0 | 0 | 0 | 0 | 0 |
| Macaca fascicularis | 1 | 2 | 3 | 0 | 0 | 1 |
| Human polypyrimidine | 1 | 0 | 0 | 0 | 0 | 3 |
| Theropithecus gelada | 1 | 1 | 0 | 0 | 3 | 5 |
| Hirundo smithii | 1 | 0 | 0 | 0 | 1 | 0 |
| Phoca vitulina | 1 | 0 | 0 | 0 | 1 | 0 |
| Myotis lucifugus | 1 | 1 | 1 | 0 | 0 | 1 |
| Pseudocnuella soli | 1 | 0 | 0 | 0 | 0 | 0 |
| Rachycentron canadum | 1 | 0 | 0 | 0 | 0 | 0 |
| Acinonyx jubatus | 1 | 0 | 0 | 0 | 0 | 0 |
| Bacillus cereus | 1 | 0 | 1 | 3 | 2 | 1 |
| Contig from | 1 | 0 | 0 | 0 | 0 | 0 |
| Uncultured organism | 1 | 0 | 0 | 0 | 0 | 0 |
| Talpa occidentalis | 1 | 0 | 0 | 0 | 0 | 0 |
| Mus musculus | 1 | 1 | 0 | 0 | 0 | 0 |
| Frigoriglobus tundricola | 1 | 0 | 0 | 0 | 0 | 0 |
| Human transforming | 1 | 0 | 0 | 0 | 0 | 0 |
| Glycine soja | 1 | 1 | 0 | 0 | 1 | 0 |
| Tursiops truncatus | 1 | 2 | 1 | 0 | 0 | 0 |
| Cloning vector | 1 | 0 | 0 | 2 | 1 | 1 |
| Pelistega ratti | 1 | 0 | 0 | 0 | 0 | 0 |
| Rhodospirillaceae bacterium | 1 | 0 | 0 | 0 | 0 | 0 |
| Hyaena hyaena | 1 | 0 | 0 | 0 | 0 | 0 |
| Mammalian expression | 1 | 1 | 1 | 0 | 1 | 2 |
| Canis lupus | 1 | 0 | 1 | 0 | 0 | 2 |
| Galeopterus variegatus | 1 | 0 | 0 | 0 | 0 | 0 |
| Human trophoblast | 1 | 0 | 1 | 0 | 0 | 0 |
| Uncultured alpha | 1 | 0 | 0 | 0 | 0 | 0 |
| Odobenus rosmarus | 1 | 0 | 0 | 0 | 1 | 0 |
| Callithrix jacchus | 1 | 1 | 0 | 1 | 0 | 0 |
| Callorhinchus milii | 1 | 0 | 0 | 0 | 0 | 0 |
| Rhinopithecus bieti | 0 | 3 | 1 | 0 | 1 | 1 |
| Colobus angolensis | 0 | 2 | 1 | 1 | 0 | 1 |
| Danio aesculapii | 0 | 2 | 1 | 0 | 0 | 1 |
| Macaca nemestrina | 0 | 1 | 0 | 1 | 1 | 1 |
| Erithacus rubecula | 0 | 1 | 0 | 0 | 1 | 0 |
| Cebus imitator | 0 | 1 | 0 | 0 | 0 | 1 |
| Arvicanthis niloticus | 0 | 1 | 0 | 0 | 0 | 0 |
| Dendrobium catenatum | 0 | 1 | 0 | 0 | 1 | 0 |
| Microcebus murinus | 0 | 1 | 0 | 0 | 0 | 0 |
| Parus major | 0 | 1 | 0 | 0 | 0 | 0 |
| Urocitellus parryii | 0 | 1 | 0 | 0 | 0 | 0 |
| Saimiri boliviensis | 0 | 1 | 1 | 0 | 2 | 0 |
| Hylobates pileatus | 0 | 1 | 0 | 0 | 0 | 0 |
| Camelus ferus | 0 | 1 | 0 | 0 | 0 | 0 |
| Mandrillus leucophaeus | 0 | 1 | 0 | 0 | 1 | 0 |
| Human clone | 0 | 0 | 0 | 0 | 0 | 1 |
| Aotus nancymaae | 0 | 0 | 1 | 0 | 2 | 1 |
| Mastomys coucha | 0 | 0 | 1 | 0 | 0 | 1 |
| Grammomys surdaster | 0 | 0 | 0 | 0 | 0 | 1 |
| Human alpha | 0 | 0 | 0 | 0 | 0 | 1 |
| Salarias fasciatus | 0 | 0 | 0 | 0 | 0 | 1 |
| Rhinolophus ferrumequinum | 0 | 0 | 0 | 0 | 0 | 1 |
| Nannospalax galili | 0 | 0 | 0 | 0 | 0 | 1 |
| Lipotes vexillifer | 0 | 0 | 0 | 0 | 0 | 1 |
| Vicugna pacos | 0 | 0 | 1 | 0 | 0 | 1 |
| Sciurus vulgaris | 0 | 0 | 1 | 1 | 0 | 1 |
| Zalophus californianus | 0 | 0 | 0 | 0 | 0 | 1 |
| Cercocebus atys | 0 | 0 | 0 | 2 | 0 | 1 |
| Human mitosin | 0 | 0 | 0 | 0 | 0 | 1 |
| Clostridium botulinum | 0 | 0 | 0 | 0 | 0 | 1 |
| Phodopus sungorus | 0 | 0 | 1 | 0 | 0 | 0 |
| Human liver | 0 | 0 | 1 | 0 | 0 | 0 |
| Triticum aestivum | 0 | 0 | 1 | 0 | 0 | 0 |
| Rattus norvegicus | 0 | 0 | 1 | 0 | 1 | 0 |
| Human transcription | 0 | 0 | 1 | 0 | 0 | 0 |
| Phocoena sinus | 0 | 0 | 1 | 0 | 0 | 0 |
| Chlorocebus sabaeus | 0 | 0 | 1 | 1 | 1 | 0 |
| Human fructose | 0 | 0 | 1 | 0 | 0 | 0 |
| Oryctolagus cuniculus | 0 | 0 | 1 | 0 | 0 | 0 |
| Contains the | 0 | 0 | 0 | 0 | 2 | 0 |
| Rousettus aegyptiacus | 0 | 0 | 0 | 0 | 1 | 0 |
| Pipistrellus kuhlii | 0 | 0 | 0 | 0 | 1 | 0 |
| Bos taurus | 0 | 0 | 0 | 0 | 1 | 0 |
| Human hereditary | 0 | 0 | 0 | 0 | 1 | 0 |
| Human heparan | 0 | 0 | 0 | 0 | 1 | 0 |
| Acomys russatus | 0 | 0 | 0 | 2 | 0 | 0 |
| Human oligodendrocyte | 0 | 0 | 0 | 1 | 0 | 0 |
| Dipodomys ordii | 0 | 0 | 0 | 1 | 0 | 0 |
| Artibeus jamaicensis | 0 | 0 | 0 | 1 | 0 | 0 |
| Human ferritin | 0 | 0 | 0 | 1 | 0 | 0 |
| Otolemur garnettii | 0 | 0 | 0 | 1 | 0 | 0 |
| Cutibacterium acnes | 0 | 0 | 0 | 1 | 0 | 0 |
| Sirthenea ocularis | 0 | 0 | 0 | 1 | 0 | 0 |
| Choloepus didactylus | 0 | 0 | 0 | 1 | 0 | 0 |
| Contains six | 0 | 0 | 0 | 1 | 0 | 0 |
